# The stress hyperglycemia ratio as a novel risk marker for postoperative delirium after cardiac valve surgery

**DOI:** 10.1038/s41598-026-41714-w

**Published:** 2026-03-02

**Authors:** Lin Zhang, Xing Zhang, Qing Wang, Li Yin, Yang Zhang, Yao Fan, LiuBao Gu, Jie Chen, Bo Gui

**Affiliations:** 1 Department of Anesthesiology and Perioperative Medicine, The First Affiliated Hospital of Nanjing Medical University, Nanjing, China; 2 Department of Anesthesiology and Pain Medicine, Geriatric Hospital of Nanjing Medical University, Nanjing, Jiangsu China; 3 Division of Clinical Epidemiology, Geriatric Hospital of Nanjing Medical University, Nanjing, China; 4 Department of Neurology, The Second Affiliated Hospital of Nanjing Medical University, Nanjing, Jiangsu China

**Keywords:** Stress hyperglycemia ratio, Postoperative delirium, Cardiac valve surgery, Prognosis, Cardiology, Diseases, Medical research, Risk factors

## Abstract

The stress hyperglycemia ratio (SHR) has been demonstrated to be associated with numerous adverse outcomes; however, its relationship with postoperative delirium (POD) in patients undergoing cardiac valve surgery remains unclear. This study aimed to investigate the association between SHR and POD in this surgical population. This retrospective study analyzed the data from 1830 adult patients who underwent cardiac valve surgery from the Medical Information Mart for Intensive Care IV (MIMIC-IV, version 3.1) database. Primary outcome was the incidence of POD within 7 postoperative days. Secondary outcomes included lengths of stay in the intensive care unit (ICU) and hospital, as well as 28-day and 90-day mortality. Multivariable logistic regression identified SHR as an independent risk factor for POD (odds ratio [OR] 1.47, 95% confidence interval [CI] 1.03–2.11, *P* = 0.034). Using the optimal SHR cutoff, patients were stratified into high-SHR (≥ 1.164) and low-SHR (< 1.164) groups. Those with high SHR had a significantly elevated risk of POD compared to the low-SHR group (OR 1.55, 95% CI 1.18–2.03, *P* = 0.002). Sensitivity analyses confirmed the robustness of these findings. After 1:1 propensity score matching based on key confounders, the high-SHR group also exhibited prolonged ICU stay and higher 28-day and 90-day mortality. No significant interactions were detected in any of the predefined subgroup analyses. These findings suggest that SHR is an important risk factor for POD following cardiac valve surgery and demonstrates considerable potential as a novel risk marker for POD in this surgical population.

**Supplementary Information:**

The online version contains supplementary material available at 10.1038/s41598-026-41714-w.

## Introduction

Postoperative delirium (POD) is a common neurocognitive complication following surgery, characterized by acute disturbances in attention, awareness, and cognition^1^. Its incidence is markedly elevated after cardiac surgery, with reported rates ranging from 11.3% to 51.6%^2^. POD is associated with prolonged hospital stays, increased mortality, and potential long-term cognitive impairment^3–5^. Consequently, the identification of significant risk factors and predictors is crucial for facilitating prevention and improving clinical management strategies.

The stress hyperglycemia ratio (SHR), defined as the ratio of admission blood glucose (BG) to chronic average BG, quantifies acute glucose fluctuations and metabolic dysregulation during physiologic stress^6^. It offers a significant advantage over isolated BG measurements by integrating baseline glycemic status, thereby providing a more precise depiction of the acute stress response^7^. Although various glucose-related markers—such as preoperative BG and mean absolute BG—have been linked to POD risk^8,9^, conventional parameters exhibit limited ability in reflecting dynamic dysregulation during surgical stress. In contrast, SHR is better suited to this surgical stress context. Nonetheless, the link between SHR and POD, especially in the cardiac valve surgery population, remains unclear and warrants further investigation.

This study was designed not only to examine the association between SHR and POD in patients undergoing cardiac valve surgery but also to explore its clinical relevance by evaluating its potential as a risk marker for risk stratification and preventive interventions.

## Materials and methods

### Source of data

This retrospective study utilized data from the Medical Information Mart for Intensive Care IV (MIMIC-IV, version 3.1) database, a publicly available clinical database. MIMIC-IV contains de-identified health records of patients admitted to the Beth Israel Deaconess Medical Center (Boston, MA, USA) between 2008 and 2019, including comprehensive information on demographics, diagnoses, laboratory results, surgical procedures, medications, vital signs, and clinical outcomes. Approval for the use of the MIMIC-IV database was obtained from the Institutional Review Boards of the Massachusetts Institute of Technology and Beth Israel Deaconess Medical Center, which also waived the requirement for informed consent due to the de-identified nature of the data. One author completed the required Collaborative Institutional Training Initiative (CITI) program and passed the associated qualifying examination (Certification Record ID: 68258918) to obtain data access. All methods were performed in accordance with relevant guidelines and regulations, and the study adhered to the principles of the Declaration of Helsinki. This study was reported in line with the Strengthening the Reporting of Observational Studies in Epidemiology (STROBE) guidelines.

### Patient selection

The study cohort comprised adult patients (aged 18–100 years) who underwent cardiac valve surgery (identified by International Classification of Diseases [ICD]-9 and ICD-10 codes; Supplementary Table 1) with subsequent postoperative intensive care unit (ICU) admission. We excluded patients with a history of schizophrenia, pre-existing dementia, or preoperative delirium. Additionally, we excluded those not admitted to the ICU for the first time during the hospitalization, with an ICU stay of less than 24 h, with missing data for glycated hemoglobin (HbA1c) or admission BG, without POD assessment, or with missing data on type of surgery.

### Study variables

Data extraction was performed from the MIMIC-IV database using Structured Query Language (SQL) via Navicat Premium (version 16.1.15). The collected variables encompassed the following domains: (1) demographic information: age, sex, ethnicity, and body mass index (BMI); (2) comorbidities: hypertension, diabetes mellitus, myocardial infarction, congestive heart failure, cerebrovascular disease, chronic pulmonary disease, chronic liver disease, chronic kidney disease, and malignant cancer; (3) surgery-related information: type of cardiac valve surgery, specific valve(s) involved, and use of cardiopulmonary bypass (CPB); (4) initial laboratory findings obtained on the first ICU day: hemoglobin, serum albumin, sodium, potassium, blood urea nitrogen (BUN), serum creatinine, BG, and HbA1c; (5) mean vital signs on the first ICU day: heart rate, systolic blood pressure (SBP), diastolic blood pressure (DBP), mean arterial pressure (MAP), peripheral oxygen saturation (SpO₂), and respiratory rate; (6) disease severity scores: Acute Physiology Score III (APS III), maximum Sequential Organ Failure Assessment (SOFA) score, and Charlson Comorbidity Index (CCI); (7) ICU medication administration: use of vasoactive drugs and sedatives; (8) clinical management: receipt of renal replacement therapy (RRT) and mechanical ventilation; (9) clinical outcomes: incidence of delirium within the first 7 postoperative days, the frequency of POD assessments, lengths of ICU and hospital stay, and 28-day and 90-day all-cause mortality.

The primary exposure was the SHR, a metric that quantifies acute glycemic fluctuations in response to physiologic stress. SHR was calculated using the formula: *SHR = blood glucose max (mg/dL) / [28.7 × HbA1c (%) – 46.7]*, as previously validated^10^. Maximum BG refers to the highest value recorded during the initial 24-hour postoperative period. If an HbA1c measurement was not available within 24 h following ICU admission, the first value obtained during the hospitalization was used.

### Primary outcome

The primary outcome was the incidence of POD within the first 7 days after cardiac valve surgery. POD was ascertained using the Confusion Assessment Method for the Intensive Care Unit (CAM-ICU) within the MIMIC-IV database. The delirium assessment protocol began with evaluating consciousness levels using the Richmond Agitation-Sedation Scale (RASS), and only patients who scored − 3 or higher proceeded to formal CAM-ICU evaluation. A diagnosis of delirium according to the CAM-ICU requires the presence of four features: (1) acute change or fluctuating mental status (MIMIC-IV item IDs: 228337, 228300, 229326); (2) inattention (IDs: 228301, 229325, 228336); (3) altered level of consciousness (ID: 228334); and (4) disorganized thinking (IDs: 228335, 228303, 229324). The coexistence of features 1 and 2, plus either feature 3 or 4, constituted a positive delirium diagnosis. Secondary outcomes included the durations of ICU and hospital stays, alongside 28-day and 90-day all-cause mortality.

### Statistical analysis

All statistical analyses were performed using R software (version 4.5.1; http://www.R-project.org, The R Foundation). Patients with missing data on HbA1c, BG, or type of surgery were excluded from the analysis. Variables with more than 20% missing values were also excluded. For remaining variables containing ≤ 20% missing data, multiple imputation was implemented using the mice package with a random forest algorithm^11^. The distribution of continuous variables was assessed for normality. Normally distributed variables are summarized as mean ± standard deviation and compared using the Student’s *t*-test; non-normally distributed variables are reported as median (interquartile range, IQR) and compared with the Mann–Whitney *U* test. Categorical variables are expressed as number (percentage) and compared using the chi-square test or Fisher’s exact test, as appropriate.

Covariates were selected via least absolute shrinkage and selection operator (LASSO) regression, supplemented by clinically relevant predictors. The association between SHR and POD was evaluated using multivariate logistic regression. Multicollinearity was assessed by calculating variance inflation factors (VIF), with variables exhibiting VIF > 5 considered for exclusion. Four sequential models were constructed. Model 1: Unadjusted. Model 2: Adjusted for demographic factors (age, sex, BMI). Model 3: Further adjusted for preoperative comorbidities and disease severity scores (diabetes, cerebrovascular disease, CCI, APS III, SOFA score). Model 4: Additionally adjusted for surgical and treatment-related variables (hemoglobin, respiratory rate, specific valve(s) involved, type of surgery, use of vasoactive drugs, sedatives, RRT, and mechanical ventilation). Model discrimination was evaluated using the area under the curve (AUC), with internal validation via 1,000 bootstrap samples. The optimal SHR cutoff for predicting POD was determined from the receiver operating characteristic (ROC) curve using the Youden index. Patients were subsequently stratified into high- and low-SHR groups based on this threshold, and a logistic regression model was applied to evaluate POD risk in the high-SHR group, using the low-SHR group as reference. To improve clinical interpretability, we calculated the odds ratio (OR) per 0.1-unit SHR increment. To assess the added value of SHR beyond conventional severity scores, we computed the continuous Net Reclassification Improvement (cNRI) for models incorporating SHR in addition to the APS III or SOFA scores. The sample size was validated post hoc using the events per variable (EPV) criterion. With an EPV of 10 and 17 predictor variables, a minimum of 170 POD events was required. This study included 326 POD events, substantially exceeding this threshold and supporting the reliability of the results.

To evaluate the robustness of our primary findings, we conducted a series of sensitivity analyses: (1) adjusting for antidiabetic medications (including insulin) to account for potential confounding effects on postoperative glucose homeostasis; (2) excluding patients diagnosed with postoperative sepsis to minimize confounding from severe systemic infection and its associated neurological complications; (3) re-analyzing the data after removing the top and bottom 1% of SHR values to assess the impact of extreme observations; (4) treating SHR as a categorical variable by dividing it into tertiles in multivariate logistic regression models.

Subgroup analyses were conducted to examine the consistency of the SHR–POD association across predefined subgroups: age (18–60, 60–75, ≥ 75 years), sex, BMI (< 28 vs. ≥28 kg/m²), diabetes, cerebrovascular disease, congestive heart failure, type of surgery (open, percutaneous, or transapical), and use of RRT. Interaction effects were tested via likelihood ratio tests, with *P*-values for interaction reported.

For the comparison of other postoperative outcomes between the low-SHR group and the high-SHR group, propensity score matching (PSM) was performed using a 1:1 nearest-neighbor algorithm with a caliper of 0.2, based on key confounding variables. These key confounding variables were determined as those showing statistically significant differences in baseline characteristics between the two SHR groups. Outcomes were compared in the matched cohort. A two-tailed *P*-value < 0.05 was considered statistically significant for all analyses.

## Results

### Study participants and baseline characteristics

**Fig. 1:**
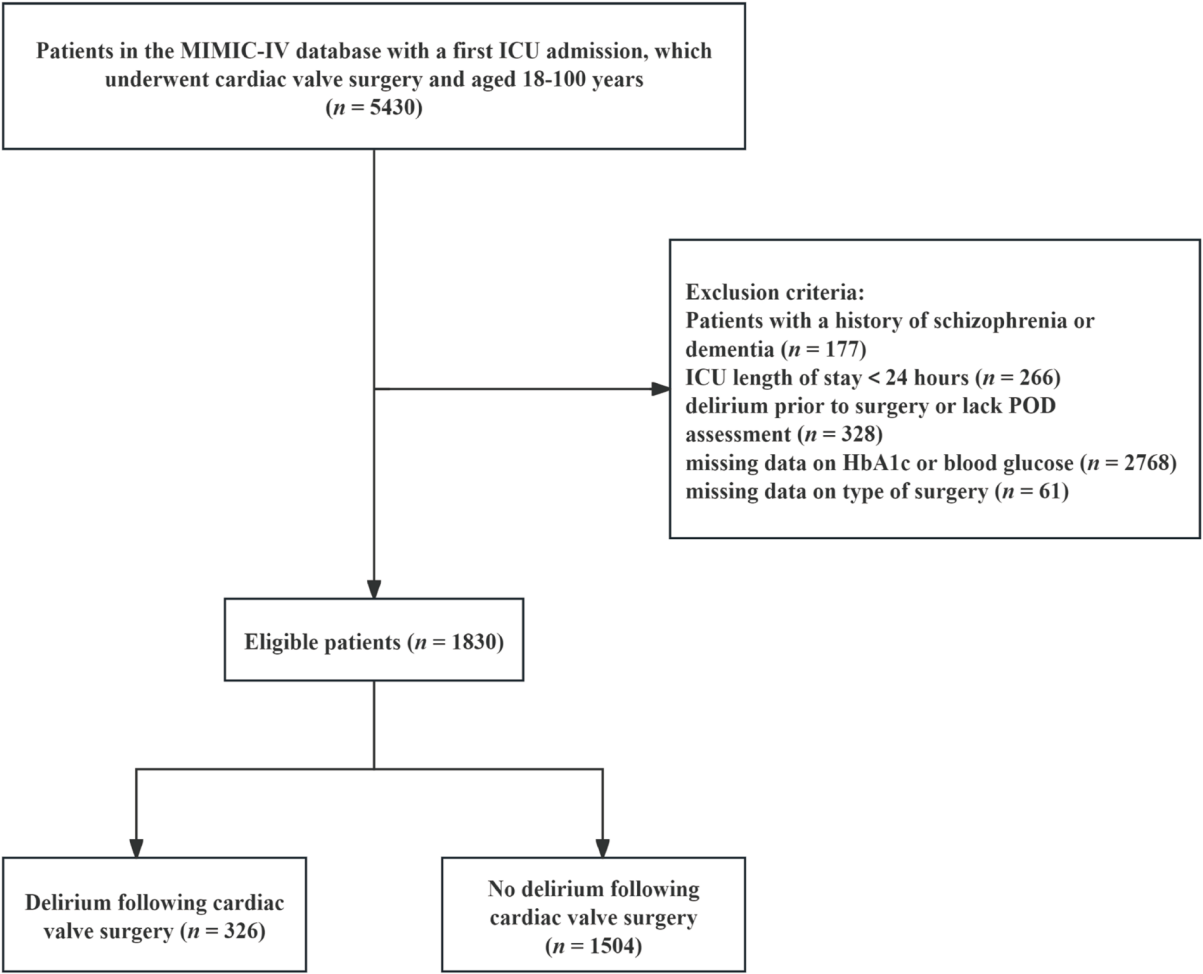
Flowchart of included patients from the MIMIC-IV database.

Figure 1 illustrates the patient selection process. A total of 1,830 patients who underwent cardiac valve surgery were included, of whom 1,144 (62.5%) were male and 686 (37.5%) were female. POD occurred in 326 patients (17.8%) within 7 days after surgery. Over the first seven postoperative days, the frequency of delirium assessments was generally comparable across days, with a modest increase observed on postoperative day 2 (Supplementary Fig. 1). As summarized in Table 1, compared to non-POD patients, those who developed POD were older and had a higher prevalence of comorbidities such as myocardial infarction, congestive heart failure, cerebrovascular disease, chronic kidney disease, and diabetes. The POD group also demonstrated higher CCI, APS III, and SOFA scores, along with lower postoperative hemoglobin levels and elevated BG and SHR values. Furthermore, POD was more frequently observed in patients undergoing multiple valve or percutaneous procedures. With respect to medications and interventions, patients with POD more commonly received vasoactive drugs, sedatives, mechanical ventilation, and RRT. Clinically, the occurrence of POD was associated with prolonged ICU and hospital stays, as well as higher 90-day mortality.

**Table 1:** Patient demographics and baseline characteristics.

Variables	Non-POD (*n* = 1504)	POD (*n* = 326)	*P* value
Age (years)	70.00 (61.00, 78.00)	72.50 (64.00, 79.75)	0.001
Sex (%)			
Female	552 (36.70)	134 (41.10)	0.154
Male	952 (63.30)	192 (58.90)	
Race (%)			
Asian	29 (1.93)	6 (1.84)	0.954
Black	52 (3.46)	14 (4.29)	
Hispanic	39 (2.59)	9 (2.76)	
White	1106 (73.54)	235 (72.09)	
Other	278 (18.48)	62 (19.02)	
BMI (kg/m^2^)	28.60 (25.19, 32.60)	28.97 (25.36, 33.98)	0.281
Myocardial Infarction (%)	259 (17.22)	81 (24.85)	0.002
Congestive heart failure (%)	699 (46.48)	198 (60.74)	< 0.001
Cerebrovascular disease (%)	162 (10.77)	62 (19.02)	< 0.001
Hypertension (%)	870 (57.85)	167 (51.23)	0.034
Diabetes mellitus (%)	391 (26.00)	124 (38.04)	< 0.001
Chronic pulmonary disease (%)	401 (26.66)	87 (26.69)	1.000
Chronic kidney disease (%)	283 (18.82)	96 (29.45)	< 0.001
Chronic liver disease (%)	86 (5.72)	17 (5.21)	0.822
Malignant cancer (%)	42 (2.79)	9 (2.76)	1.000
CCI	4.00 (3.00, 6.00)	5.00 (4.00, 7.00)	< 0.001
Hemoglobin (g/dL)	9.40 (8.20, 10.90)	9.10 (7.70, 10.78)	0.005
Sodium (mmol/L)	138.00 (136.00, 140.00)	139.00 (136.00, 141.00)	0.042
Potassium (mmol/L)	4.30 (4.00, 4.60)	4.20 (3.90, 4.60)	0.465
BUN (mg/dL)	16.00 (13.00, 22.00)	19.00 (14.00, 26.00)	< 0.001
Creatinine (mg/dL)	0.90 (0.70, 1.10)	1.00 (0.80, 1.40)	< 0.001
Blood glucose (mg/dL)	123.00 (107.00, 142.00)	129.00 (111.25, 153.75)	< 0.001
HbA1c (%)	5.70 (5.40, 6.20)	5.80 (5.40, 6.50)	0.014
SHR	1.04 (0.88, 1.22)	1.11 (0.88, 1.33)	0.009
HR (beats/minute)	79.83 (74.77, 85.56)	81.00 (75.23, 87.90)	0.026
SBP (mmHg)	110.69 (105.25, 116.67)	109.41 (103.41, 115.81)	0.003
DBP (mmHg)	56.84 (52.29, 61.49)	55.21 (49.82, 60.99)	0.006
MAP (mmHg)	73.66 (69.88, 77.79)	72.19 (68.50, 76.98)	0.002
Respiratory rate (beats/minute)	17.72 (16.25, 19.48)	18.44 (16.61, 20.40)	< 0.001
SpO2 (%)	97.85 (96.84, 98.79)	98.05 (96.88, 98.91)	0.071
APS Ⅲ	35.00 (27.00, 46.00)	43.00 (33.00, 58.00)	< 0.001
SOFA score	6.00 (4.00, 8.00)	7.00 (5.00, 9.00)	< 0.001
Specific valve(s) involved (%)			
Aortic valve	836 (55.59)	165 (50.61)	< 0.001
Mitral valve	445 (29.59)	79 (24.23)	
Tricuspid valve	35 (2.33)	11 (3.37)	
Multiple valves	188 (12.50)	71 (21.78)	
Type of surgery (%)			
Open	1434 (95.35)	299 (91.72)	0.027
Percutaneous	60 (3.99)	24 (7.36)	
Transapical	10 (0.66)	3 (0.92)	
CPB (%)	1397 (92.89)	294 (90.18)	0.120
Use of vasoactive drugs (%)	1046 (69.55)	262 (80.37)	< 0.001
Use of sedatives (%)	1305 (86.77)	310 (95.09)	< 0.001
Mechanical ventilation (%)	1187 (78.92)	301 (92.33)	< 0.001
RRT (%)	62 (4.12)	53 (16.26)	< 0.001
Lengths of hospital stay (days)	8.92 (6.25, 12.97)	14.00 (8.38, 23.16)	< 0.001
Lengths of ICU stay (days)	2.21 (1.32, 3.45)	4.98 (2.66, 10.97)	< 0.001
28-day mortality (%)	33 (2.19)	13 (3.99)	0.093
90-day mortality (%)	54 (3.59)	31 (9.51)	< 0.001

POD, postoperative delirium; BMI, body mass index; CCI, Charlson Comorbidity Index; BUN, blood urea nitrogen; HbA1c, glycated hemoglobin; SHR, stress hyperglycemia ratio; HR, heart rate; SBP, systolic blood pressure; DBP, diastolic blood pressure; MAP, mean arterial pressure; SpO2, peripheral oxygen saturation; APS Ⅲ, Acute Physiology Score III; SOFA, Sequential Organ Failure Assessment; CPB, cardiopulmonary bypass; RRT, renal replacement therapy.

### Association between SHR and POD incidence

**Fig. 2:**
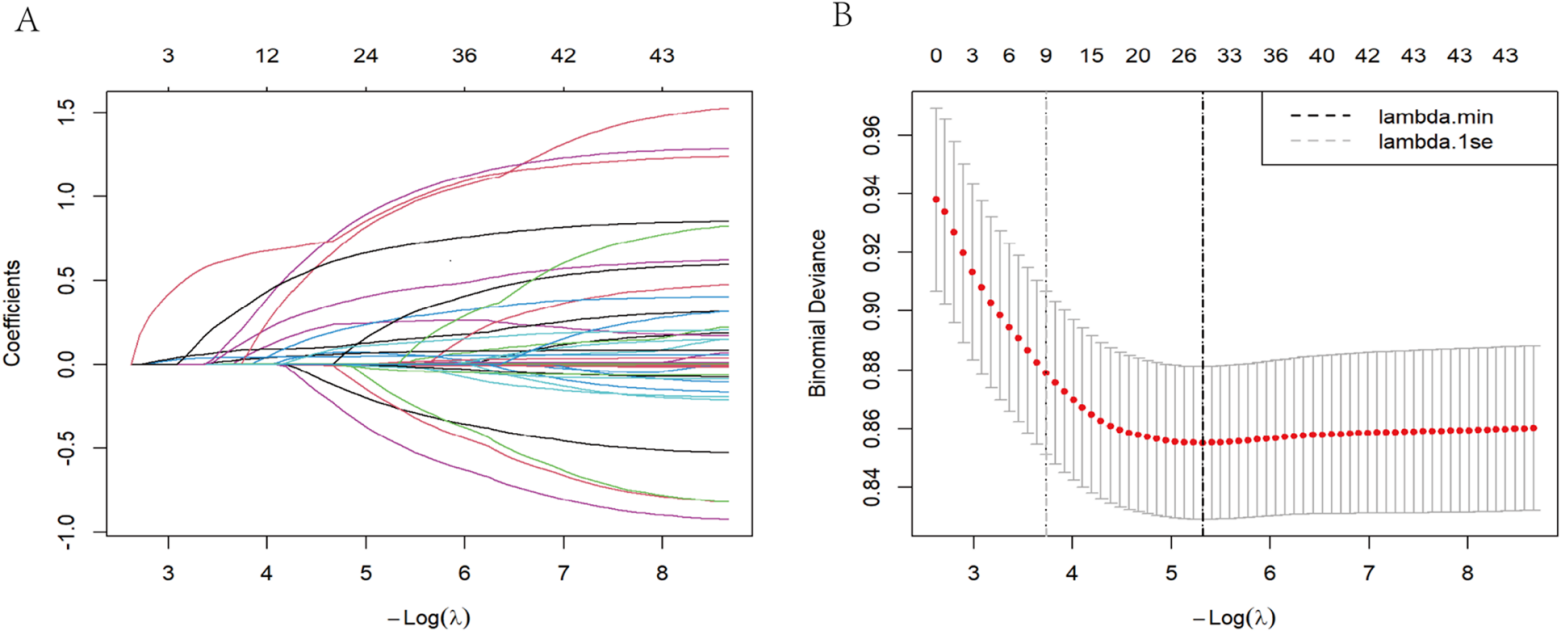
The lasso regression for variable screening. (**A**) Variation characteristics of variable coefficients; (**B**) Selection of the regularization parameter lambda (λ) was determined by cross-validation method. The grey dashed line indicates λ 1se, following the 1se rule to yield a more robust and simplified model.

Based on the lambda.1se criterion, nine non-zero coefficient variables were identified (Fig. 2). These variables were subsequently integrated with key clinical predictors of POD, resulting in a final set of 17 covariates for inclusion in the multivariate logistic regression models: SHR, age, sex, BMI, diabetes, cerebrovascular disease, CCI, APS III, SOFA score, hemoglobin, respiratory rate, specific valve(s) involved, type of surgery, use of vasoactive drugs, use of sedatives, RRT, and mechanical ventilation. All VIF values were below 5, indicating no substantial multicollinearity concerns (Supplementary Table 2).

A series of four multivariate logistic regression models (Models 1–4) were constructed with sequential adjustment for demographic characteristics, preoperative comorbidities, surgical details, and medication or therapeutic interventions. As summarized in Table 2, SHR was identified as an independent risk factor for POD after cardiac valve surgery. Each one-unit increase in SHR was associated with a 47% elevation in the odds of POD in the fully adjusted model (odds ratio [OR] 1.47, 95% confidence interval [CI] 1.03–2.11, *P* = 0.034). The final logistic regression model showed an AUC of 0.725. After internal validation with bootstrapping (1,000 replicates), the corrected AUC was 0.705, indicating stable discrimination. To quantify the effect size of a clinically relevant degree of change, we analyzed SHR per 0.1-unit increment. When analyzed per 0.1-unit increment, SHR remained a significant risk indicator of POD (adjusted OR = 1.04, 95% CI: 1.00–1.08, *P* = 0.035). To avoid multicollinearity, SHR was not included in the multivariable logistic regression models simultaneously with BG or HbA1c. When BG or HbA1c was substituted for SHR in Model 4, neither conventional marker showed a statistically or clinically meaningful association with POD comparable to that of SHR (BG: OR 1.00, 95% CI 1.00–1.01, *P* = 0.029; HbA1c: OR 1.01, 95% CI 0.88–1.16, *P* = 0.917). Other factors significantly associated with POD included a history of cerebrovascular disease, elevated respiratory rate, multiple valve surgery, percutaneous approach, use of sedatives, RRT, and mechanical ventilation (Supplementary Table 3).

**Table 2:** Relationships between SHR and the risk of POD according to different models.

Variable	Model 1		Model 2		Model 3		Model 4	
	OR (95% CI)	*P* value	OR (95% CI)	*P* value	OR (95% CI)	*P* value	OR (95% CI)	*P* value
Continuous	1.63 (1.19, 2.24)	0.002	1.74 (1.26, 2.38)	< 0.001	1.60 (1.16, 2.22)	0.005	1.47 (1.03, 2.11)	0.034
Categorical								
Low-SHR	1(ref)		1(ref)		1(ref)		1(ref)	
High-SHR	1.56 (1.22, 1.99)	< 0.001	1.61 (1.26, 2.06)	< 0.001	1.61 (1.25, 2.09)	< 0.001	1.55 (1.18, 2.03)	0.002

low-SHR: SHR < 1.164; high-SHR: SHR ≥ 1.164.

Model 1: adjusted for none.

Model 2: adjusted for Age, Sex, BMI.

Model 3: adjusted for Model 2 + Diabetes, Cerebrovascular disease, CCI, APS III, and SOFA score.

Model 4: adjusted for Model 3 + Hemoglobin, Respiratory rate, Specific valve(s) type, Type of surgery, Use of vasoactive drugs, Use of sedatives, RRT, and Mechanical ventilation.

SHR, stress hyperglycemia ratio; POD, postoperative delirium; BMI, body mass index; CCI, Charlson Comorbidity Index; APS III, Acute Physiology Score III; SOFA, Sequential Organ Failure Assessment; RRT, renal replacement therapy.

The optimal SHR cutoff for POD prediction was determined through ROC curve analysis, with the maximum Youden index identifying a value of 1.164. This cutoff corresponded to a sensitivity of 42.6%, a specificity of 68.1%, and a Youden index of 0.107. Using this threshold, patients were stratified into low-SHR (< 1.164) and high-SHR (≥ 1.164) groups. When comparing the low and high SHR groups, only minimal differences in assessment frequency were observed across postoperative days (range: 0.1–0.2 assessments/day) (Supplementary Table 4). The delirium screening intensity during the ICU stay was comparable between the two groups. As shown in Table 2, logistic regression analysis demonstrated a significantly higher risk of POD in the high-SHR group compared to the low-SHR group (OR 1.55, 95% CI 1.18–2.03, *P* = 0.002), supporting the utility of this cutoff for POD risk stratification in clinical practice.

### The incremental effect of the SHR

The predictive value of SHR beyond established clinical risk scores was evaluated by calculating the cNRI, as shown in Table 3. Adding SHR to the APS III score significantly improved reclassification, whether treated as a continuous variable (NRI = 0.140, 95% CI: 0.010–0.262, *P* = 0.038) or as a binary variable based on the optimal cutoff (NRI = 0.205, 95% CI: 0.080–0.323, *P* < 0.001). Similarly, significant improvement was observed when SHR was added to the SOFA score (SHR continuous: NRI = 0.163, 95% CI: 0.037–0.283, *P* = 0.010; SHR binary: NRI = 0.204, 95% CI: 0.080–0.322, *P* < 0.001). These results confirm that SHR provides incremental predictive value beyond conventional severity scores.

**Table 3:** Incremental effect of SHR for POD.

Score	NRI [95% CI] (+ SHR (continuous))	*P* value	NRI [95% CI] (+ SHR (binary))	*P* value
APS III	0.140 (0.010, 0.262)	0.038	0.205 (0.080, 0.323)	< 0.001
SOFA	0.163 (0.037, 0.283)	0.010	0.204 (0.080, 0.322)	< 0.001

SHR, stress hyperglycemia ratio; POD, postoperative delirium; APS III, Acute Physiology Score III; SOFA, Sequential Organ Failure Assessment; NRI, Net Reclassification Improvement.

### Sensitivity analysis

To assess the robustness of our primary findings, we conducted multiple sensitivity analyses (Supplementary Tables 5–8). In the first sensitivity analysis, which incorporated antidiabetic medications (including insulin) as a covariate while adjusting for other confounders, SHR remained significantly associated with POD (OR 1.46, 95% CI 1.02–2.09, *P* = 0.040). Antidiabetic medication use was independently associated with a reduced risk of POD (OR 0.53, 95% CI 0.31–0.91, *P* = 0.021). Second, after excluding patients who developed postoperative sepsis, SHR continued to demonstrate a significant association with POD (OR 1.51, 95% CI 1.05–2.19, *P* = 0.028). Third, to evaluate the influence of extreme values, we repeated the analysis after removing the top and bottom 1% of SHR values; the association remained robust (OR 1.66, 95% CI 1.07–2.58, *P* = 0.024). Fourth, when SHR was analyzed as a categorical variable by tertiles, patients in the highest tertile (T3, SHR > 1.167) exhibited a significantly increased risk of POD compared to those in the lowest tertile (T1, SHR < 0.943) (T3 vs. T1: OR 1.51, 95% CI 1.10–2.08, *P* = 0.012). No statistically significant difference in POD risk was observed between the middle (T2, SHR 0.943–1.167) and lowest tertiles. A significant trend was observed across SHR tertiles (*P* for trend = 0.009), further supporting a consistent dose–response relationship between SHR and POD risk.

### Subgroup analysis

**Fig. 3:**
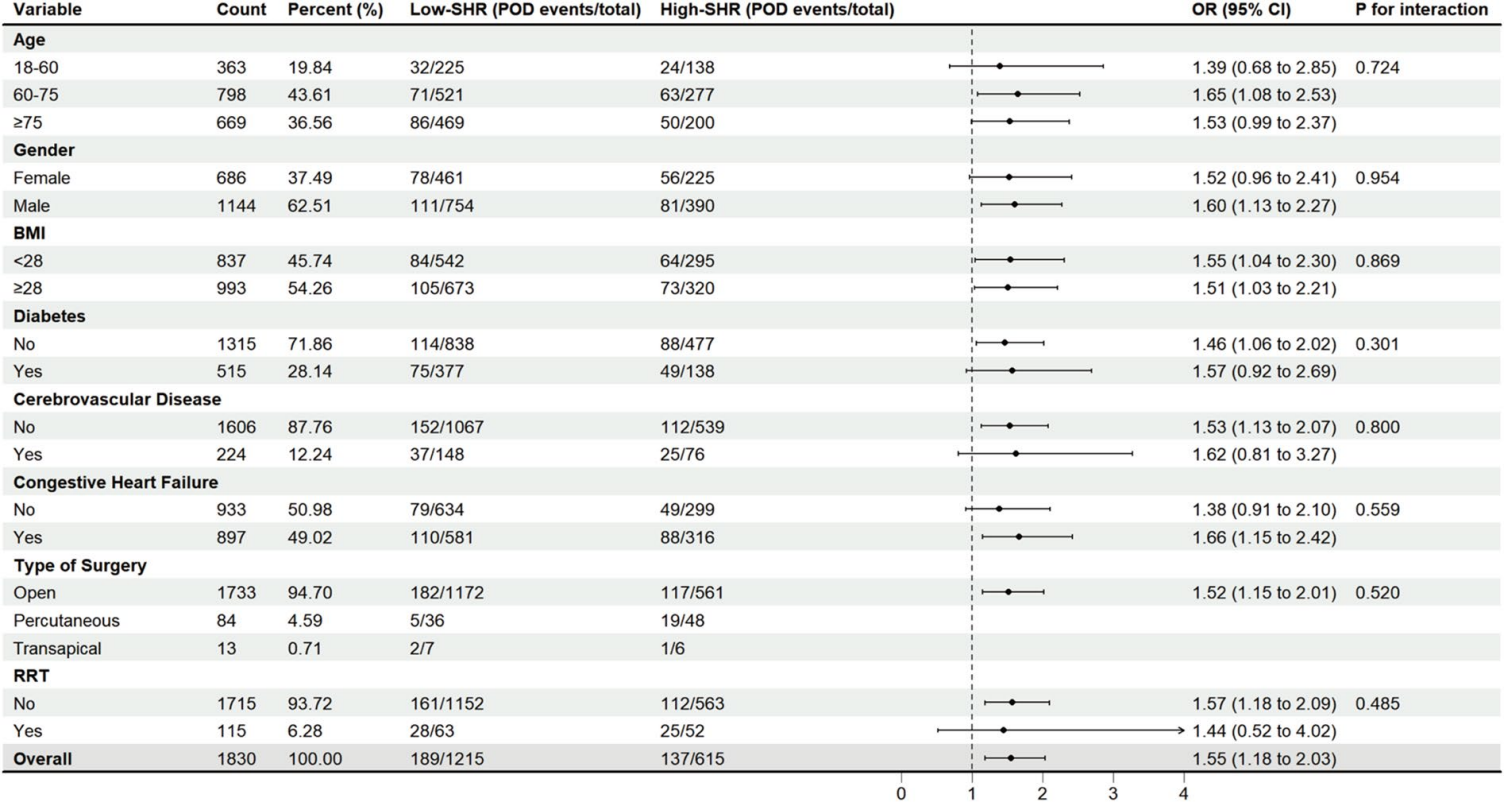
Subgroup analysis to identify variables potentially modulating the association between SHR and POD following cardiac valve surgery. low-SHR: SHR < 1.164; high-SHR: SHR ≥ 1.164. Adjusted for age, sex, BMI, diabetes, cerebrovascular disease, CCI, APS Ⅲ, SOFA score, hemoglobin, respiratory rate, specific valve(s) type, type of surgery, use of vasoactive drugs, use of sedatives, RRT, and mechanical ventilation. SHR, stress hyperglycemia ratio; POD, postoperative delirium; BMI, body mass index; CCI, Charlson Comorbidity Index; APS Ⅲ, Acute Physiology Score III; SOFA, Sequential Organ Failure Assessment; RRT, renal replacement therapy.

Patients undergoing cardiac valve surgery were categorized into low- and high-SHR groups based on an optimal cutoff value of 1.164. To evaluate potential effect modification, subgroup analyses were conducted for age, sex, BMI, diabetes, cerebrovascular disease, congestive heart failure, type of surgery, and RRT (Fig. 3). No significant interactions were detected across any subgroups (all *P* for interaction > 0.05), supporting SHR as a consistent and independent risk factor for POD in this population, with all ORs exceeding 1. Statistically significant increases in POD risk were observed in the following subgroups: patients aged 60–75 years (OR 1.65, 95% CI 1.08–2.53), males (OR 1.60, 95% CI 1.13–2.27), those with BMI < 28 kg/m^2^ (OR 1.55, 95% CI 1.04–2.30), non-diabetic patients (OR 1.46, 95% CI 1.06–2.02), patients without cerebrovascular disease (OR 1.53, 95% CI 1.13–2.07), and those not receiving RRT (OR 1.57, 95% CI 1.18–2.09). Significant associations were also noted among patients undergoing open surgery (OR 1.52, 95% CI 1.15–2.01) and those with congestive heart failure (OR 1.66, 95% CI 1.15–2.42).

### Prognosis analysis

Based on the comparison of baseline characteristics between the high-SHR and low-SHR groups (Supplementary Table 9), all variables with statistically significant differences (*P* < 0.05) were selected for inclusion in the propensity score matching, including: age, hypertension, diabetes mellitus, hemoglobin, creatinine, respiratory rate, APS Ⅲ, type of surgery, use of vasoactive drugs or sedatives, and RRT. To compare postoperative outcomes between the low-SHR and high-SHR groups while mitigating confounding, we conducted a 1:1 PSM based on key confounders. As shown in Table 4, the matched analysis revealed that the high-SHR group was associated with a significantly increased incidence of POD (*P* = 0.005), prolonged ICU length of stay (*P* = 0.007), and elevated 28-day and 90-day mortality (*P* < 0.001; *P* = 0.002).

**Table 4:** Clinical outcome for patients in different SHR categories.

	Before PSM		*P* value	After PSM		*P* value
	Low-SHR	High-SHR		Low-SHR	High-SHR	
Number	1215	615		610	610	
POD (%)	189 (15.56)	137 (22.28)	< 0.001	95 (15.57)	134 (21.97)	0.005
Lengths of hospital stay (days)	9.30(6.57, 13.93)	9.11(6.31, 15.09)	0.833	9.27 (6.33, 14.60)	9.10 (6.31, 15.06)	0.95
Lengths of ICU stay (days)	2.29(1.34, 4.1)	2.5(1.42, 4.50)	0.002	2.29 (1.34, 4.06)	2.49 (1.41, 4.46)	0.007
28-day mortality (%)	14 (1.15)	32 (5.20)	< 0.001	6 (0.98)	31 (5.08)	< 0.001
90-day mortality (%)	39 (3.21)	46 (7.48)	< 0.001	20 (3.28)	45 (7.38)	0.002

All values are presented as number (n) and proportion (%) for categorical variables, or median (interquartile range) for non-normally distributed continuous variables.

low-SHR: SHR < 1.164; high-SHR: SHR ≥ 1.164.

SHR, stress hyperglycemia ratio; PSM, propensity score matching; POD, postoperative delirium.

## Discussion

Cardiac valve surgery is associated with a high incidence of POD, reported to reach 44.6%^12^, due to prolonged operative duration, substantial surgical trauma and the frequent use of CPB during the procedure^13^, which can induce a systemic inflammatory response and greater physiological disturbance^14^ and thereby increase the risk of POD. Therefore, patients undergoing cardiac valve surgery require heightened attention for the prevention and management of POD. This study is the first, to our knowledge, to validate that an elevated SHR is a significant risk factor for POD after cardiac valve surgery. This association remained independent and significant after multivariable adjustment, underscoring the clinical relevance of SHR as a risk marker. Its consistency was affirmed by stable results in sensitivity analyses and a lack of significant interactions across all prespecified subgroups, indicating the broad applicability of our findings. Therefore, as a readily available and easily derived metric, SHR can serve as a practical risk marker for the early detection of high-risk patients, enabling timely interventions that may guide prevention and ultimately improve outcomes in cardiac surgery. While SHR is significantly associated with POD, it should be interpreted as a risk indicator rather than an independent predictive tool. SHR is best regarded as a complementary marker that can refine POD risk stratification when used in conjunction with established clinical assessments and scoring systems.

The surgical stress response, characterized by catecholamine and cortisol release, disrupts glucose homeostasis and promotes stress-induced hyperglycemia and glycemic variability^15^. These dynamic changes are not fully captured by static glycemic measures, highlighting the particular relevance of the SHR in this context. Our study is the first to establish SHR as a significant risk factor of postoperative delirium (POD) after cardiac valve surgery, addressing a key knowledge gap. We observed that each one-unit increase in SHR was associated with a nearly 50% elevation in POD risk. Given the well-documented adverse impact of POD on recovery, this substantial risk underscores the need for vigilant monitoring and early identification of patients with elevated SHR. Although the association between higher SHR and POD was statistically significant, with an adjusted odds ratio (aOR) of 1.04 per 0.1‑unit increment, this suggests that SHR should be interpreted as a modest contributor within a multifactorial risk profile rather than a strong standalone predictor. The link between stress hyperglycemia and delirium is also supported by evidence from non-surgical settings. For instance, a single-center retrospective study identified a strong association between SHR and delirium risk in elderly patients with community-acquired pneumonia^16^. Similarly, SHR remained an independent predictor of delirium in patients with heart failure^17^, collectively underscoring a consistent relationship across diverse clinical populations. Notably, our data also indicated a protective effect of antidiabetic medications, including insulin, against POD under stress conditions. This observation suggests a potential therapeutic avenue for POD prevention in high-risk patients experiencing stress-induced hyperglycemia, a promising hypothesis that merits validation in future large-scale, multicenter prospective studies.

The optimal cutoff value for the SHR varies across clinical conditions and remains to be standardized. This variation is evident in reports linking an SHR > 0.77–0.79 to increased all-cause mortality in critical cerebrovascular disease^18^, an SHR ≥ 1.30 to higher long-term mortality in critically ill patients with acute myocardial infarction^19^, and an SHR > 1.12 to a heightened risk of delirium in older adults with community-acquired pneumonia^20^. In the present study, ROC curve analysis identified 1.164 as the optimal SHR cutoff for predicting POD following cardiac valve surgery. Risk stratification based on this threshold confirmed that an SHR ≥ 1.164 is associated with an increased risk of POD, supporting its use for early identification and timely intervention. Furthermore, an SHR ≥ 1.164 was also associated with prolonged ICU stay and increased 28-day and 90-day mortality, underscoring its value as a key prognostic indicator. This broader prognostic role of SHR has been similarly established in other populations, including patients with sepsis, atrial fibrillation, or cardiovascular-kidney-metabolic syndrome^21–23^.

A well-established pathway links hyperglycemia to the activation of inflammation and oxidative stress via multiple signaling pathways, as demonstrated in vitro^24,25^. Notably, these mechanisms—inflammation and oxidative stress—are increasingly recognized as key contributors to delirium pathogenesis in various clinical settings^26–29^. This pathophysiological overlap likely underlies the correlation between a high SHR and POD identified in our study. Moreover, translational support comes from diabetic mouse models, where hyperglycemia has been shown to drive neuroinflammation, blood-brain barrier disruption, and subsequent cognitive deficits^30^. Collectively, these findings provide strong mechanistic support for our clinical results.

The rationale for using the maximum blood glucose value within 24 h postoperatively to calculate SHR is as follows: First, this approach is consistent with established methodology in the cardiac surgery literature^10^. Second, postoperative glucose levels reflect the acute physiological stress response triggered by cardiopulmonary bypass, anesthesia, and early hemodynamic fluctuations. Third, prior research has demonstrated that using maximum rather than admission glucose to compute SHR not only better captures stress-induced hyperglycemia and its association with poorer prognosis, but also avoids the need for using different thresholds for diabetic versus non-diabetic patients^31^.

We acknowledge several limitations in our study. First, this study was conducted at a single urban academic medical center (Beth Israel Deaconess Medical Center), which may limit the external generalizability of our findings. Second, although we used postoperative maximum glucose to calculate SHR in our primary analysis, this approach cannot fully exclude residual confounding or potential bidirectional effects. Third, the absence of detailed perioperative and anesthesia-specific data—including depth of anesthesia, opioid exposure, and hemodynamic management—may have introduced residual confounding. Although we adjusted for sedative use, detailed information on sedation depth and duration was not available in the database, limiting our ability to fully account for these factors. Fourth, although Troponin T is a recognized predictor of cardiogenic shock after heart valve surgery^32^, its potential association with POD could not be evaluated in this study due to critically low data completeness in the MIMIC-IV database, with missing rates exceeding 85% preoperatively and 93% postoperatively. Finally, information on delirium subtype, duration, and severity was not available, precluding further analysis of whether SHR is associated with specific delirium phenotypes or with more persistent or severe forms of delirium. In addition, the potential under-recognition of hypoactive delirium remains an inherent limitation of retrospective designs, particularly among sedated or mechanically ventilated patients. These limitations should be addressed in future multicenter prospective studies.

## Conclusion

In conclusion, this study identifies the SHR as a significant risk indicator for POD following cardiac valve surgery and shows it is correlated with increased all-cause mortality. Implementing perioperative SHR monitoring could enhance risk stratification and facilitate the timely initiation of targeted interventions for high-risk patients. However, it should be regarded as an adjunctive risk marker that complements, rather than replaces, comprehensive clinical evaluation and established risk scores.

## Supplementary Information

Below is the link to the electronic supplementary material.

Supplementary Material 1

## Data Availability

Data in this study were obtained from the MIMIC-IV database. Available at: https://physionet.org/.

## Abbreviations

APS III: Acute physiology score III
BG: Blood glucose
BUN: Blood urea nitrogen
CAM-ICU: Confusion assessment method for the intensive care unit
CCI: Charlson comorbidity index
CPB: Cardiopulmonary bypass
DBP: Diastolic blood pressure
EPV: Events per variable
HbA1c: Glycated hemoglobin
ICD: International classification of diseases
ICU: Intensive care unit
LASSO: Least absolute shrinkage and selection operator
MAP: Mean arterial pressure
MIMIC-IV: Medical information mart for intensive care IV
NRI: Net reclassification improvement
POD: Postoperative delirium
PSM: Propensity score matching
RASS: Richmond agitation-sedation scale
ROC: Receiver operating characteristic
RRT: Renal replacement therapy
SBP: Systolic blood pressure
SHR: Stress hyperglycemia ratio
SOFA: Sequential organ failure assessment
SpO₂: Peripheral oxygen saturation
SQL: Structured query language
STROBE: Strengthening the reporting of observational studies in epidemiology
VIF: Variance inflation factors
