# Supplementary material for: The stress hyperglycemia ratio as a novel risk marker for postoperative delirium after cardiac valve surgery
Source: Sci Rep. 2026 Mar 2;16:11517. doi: 10.1038/s41598-026-41714-w (PMC13057241; doi:10.1038/s41598-026-41714-w)

**Supplementary Table 1.** The ICD codes of cardiac valve surgery.

| Valve surgery | 0203,024F07J,024F08J,024F0JJ,024F0KJ,024G072,024G082,024G0J2,024G0K2,024J072,024J082,024J0J2,024J0K2,025F0ZZ,025F3ZZ,025F4ZZ,025G0ZZ,025G3ZZ,025G4ZZ,025H0ZZ,025H3ZZ,025H4ZZ,025J0ZZ,025J3ZZ,025J4ZZ,027F04Z,027F0DZ,027F0ZZ,027F34Z,027F3DZ,027F3ZZ,027F44Z,027F4DZ,027F4ZZ,027G04Z,027G0DZ,027G0ZZ,027G34Z,027G3DZ,027G3ZZ,027G44Z,027G4DZ,027G4ZZ,027H04Z,027H0DZ,027H0ZZ,027H34Z,027H3DZ,027H3ZZ,027H44Z,027H4DZ,027H4ZZ,027J04Z,027J0DZ,027J0ZZ,027J34Z,027J3DZ,027J3ZZ,027J44Z,027J4DZ,027J4ZZ,02BF0ZX,02BF0ZZ,02BF3ZX,02BF3ZZ,02BF4ZX,02BF4ZZ,02BG0ZX,02BG0ZZ,02BG3ZX,02BG3ZZ,02BG4ZX,02BG4ZZ,02BH0ZX,02BH0ZZ,02BH3ZX,02BH3ZZ,02BH4ZX,02BH4ZZ,02BJ0ZX,02BJ0ZZ,02BJ3ZX,02BJ3ZZ,02BJ4ZX,02BJ4ZZ,02CF0ZZ,02CF3ZZ,02CF4ZZ,02CG0ZZ,02CG3ZZ,02CG4ZZ,02CH0ZZ,02CH3ZZ,02CH4ZZ,02CJ0ZZ,02CJ3ZZ,02CJ4ZZ,02LH0CZ,02LH0DZ,02LH0ZZ,02LH3CZ,02LH3DZ,02LH3ZZ,02LH4CZ,02LH4DZ,02LH4ZZ,02NF0ZZ,02NF3ZZ,02NF4ZZ,02NG0ZZ,02NG3ZZ,02NG4ZZ,02NH0ZZ,02NH3ZZ,02NH4ZZ,02NJ0ZZ,02NJ3ZZ,02NJ4ZZ,02QF0ZJ,02QF0ZZ,02QF3ZJ,02QF3ZZ,02QF4ZJ,02QF4ZZ,02QG0ZE,02QG0ZZ,02QG3ZE,02QG3ZZ,02QG4ZE,02QG4ZZ,02QH0ZZ,02QH3ZZ,02QH4ZZ,02QJ0ZG,02QJ0ZZ,02QJ3ZG,02QJ3ZZ,02QJ4ZG,02QJ4ZZ,02RF07Z,02RF08N,02RF08Z,02RF0JZ,02RF0KZ,02RF37H,02RF37Z,02RF38H,02RF38N,02RF38Z,02RF3JH,02RF3JZ,02RF3KH,02RF3KZ,02RF47Z,02RF48N,02RF48Z,02RF4JZ,02RF4KZ,02RG07Z,02RG08Z,02RG0JZ,02RG0KZ,02RG37H,02RG37Z,02RG38H,02RG38Z,02RG3JH,02RG3JZ,02RG3KH,02RG3KZ,02RG47Z,02RG48Z,02RG4JZ,02RG4KZ,02RH07Z,02RH08Z,02RH0JZ,02RH0KZ,02RH37H,02RH37Z,02RH38H,02RH38L,02RH38M,02RH38Z,02RH3JH,02RH3JZ,02RH3KH,02RH3KZ,02RH47Z,02RH48Z,02RH4JZ,02RH4KZ,02RJ07Z,02RJ08Z,02RJ0JZ,02RJ0KZ,02RJ37H,02RJ37Z,02RJ38H,02RJ38Z,02RJ3JH,02RJ3JZ,02RJ3KH,02RJ3KZ,02RJ47Z,02RJ48Z,02RJ4JZ,02RJ4KZ,02TH0ZZ,02TH3ZZ,02TH4ZZ,02UF07J,02UF07Z,02UF08J,02UF08Z,02UF0JJ,02UF0JZ,02UF0KJ,02UF0KZ,02UF37J,02UF37Z,02UF38J,02UF38Z,02UF3JJ,02UF3JZ,02UF3KJ,02UF3KZ,02UF47J,02UF47Z,02UF48J,02UF48Z,02UF4JJ,02UF4JZ,02UF4KJ,02UF4KZ,02UG07E,02UG07Z,02UG08E,02UG08Z,02UG0JE,02UG0JZ,02UG0KE,02UG0KZ,02UG37E,02UG37Z,02UG38E,02UG38Z,02UG3JE,02UG3JH,02UG3JZ,02UG3KE,02UG3KZ,02UG47E,02UG47Z,02UG48E,02UG48Z,02UG4JE,02UG4JZ,02UG4KE,02UG4KZ,02UH07Z,02UH08Z,02UH0JZ,02UH0KZ,02UH37Z,02UH38Z,02UH3JZ,02UH3KZ,02UH47Z,02UH48Z,02UH4JZ,02UH4KZ,02UJ07G,02UJ07Z,02UJ08G,02UJ08Z,02UJ0JG,02UJ0JZ,02UJ0KG,02UJ0KZ,02UJ37G,02UJ37Z,02UJ38G,02UJ38Z,02UJ3JG,02UJ3JZ,02UJ3KG,02UJ3KZ,02UJ47G,02UJ47Z,02UJ48G,02UJ48Z,02UJ4JG,02UJ4JZ,02UJ4KG,02UJ4KZ,02VG0ZZ,02VG3ZZ,02VG4ZZ,02WF07Z,02WF08Z,02WF0JZ,02WF0KZ,02WF37Z,02WF38Z,02WF3JZ,02WF3KZ,02WF47Z,02WF48Z,02WF4JZ,02WF4KZ,02WG07Z,02WG08Z,02WG0JZ,02WG0KZ,02WG37Z,02WG38Z,02WG3JZ,02WG3KZ,02WG47Z,02WG48Z,02WG4JZ,02WG4KZ,02WH07Z,02WH08Z,02WH0JZ,02WH0KZ,02WH37Z,02WH38Z,02WH3JZ,02WH3KZ,02WH47Z,02WH48Z,02WH4JZ,02WH4KZ,02WJ07Z,02WJ08Z,02WJ0JZ,02WJ0KZ,02WJ37Z,02WJ38Z,02WJ3JZ,02WJ3KZ,02WJ47Z,02WJ48Z,02WJ4JZ,02WJ4KZ,3500,3501,3502,3503,3504,3505,3506,3507,3508,3509,3510,3511,3512,3513,3514,3520,3521,3522,3523,3524,3525,3526,3527,3528,3533,3539,3552,3596,3597,3599,X2RF032,X2RF332 |
| --- | --- |

**Supplementary Table 2.** Variance inflation factors for all the included variables.

| Predictor Variable | GVIF | GVIF^(1/(2*Df)) |
| --- | --- | --- |
| SHR | 1.133 | 1.065 |
| Age | 1.854 | 1.362 |
| Sex | 1.116 | 1.057 |
| BMI | 1.210 | 1.100 |
| Diabetes | 1.405 | 1.185 |
| Cerebrovascular disease | 1.130 | 1.063 |
| CCI | 2.469 | 1.571 |
| APS III | 1.890 | 1.375 |
| SOFA score | 2.057 | 1.434 |
| Hemoglobin | 1.127 | 1.062 |
| Respiratory rate | 1.192 | 1.092 |
| Specific valve(s) type | 1.206 | 1.032 |
| Type of surgery | 1.449 | 1.097 |
| Use of vasoactive drugs | 1.115 | 1.056 |
| Use of sedatives | 1.240 | 1.113 |
| RRT | 1.296 | 1.138 |
| Mechanical ventilation | 1.128 | 1.062 |

*VIF*, variance inflation factors; *SHR*, stress hyperglycemia ratio; *BMI*, body mass index; *CCI*, Charlson Comorbidity Index; *APS III*, Acute Physiology Score III; *SOFA*, Sequential Organ Failure Assessment; *RRT*, renal replacement therapy.

**Supplementary Table 3.** Logistic regression analysis of factors associated with POD

| Variable | Model 4 | |
| --- | --- | --- |
|  | OR (95% CI) | *P* value |
| SHR | 1.47 (1.03, 2.11) | 0.034 |
| Age | 1.01 (1.00, 1.03) | 0.117 |
| Sex |  |  |
| Female |  |  |
| Male | 0.94 (0.71, 1.24) | 0.654 |
| BMI | 1.01 (0.99, 1.03) | 0.472 |
| Diabetes mellitus |  |  |
| No |  |  |
| Yes | 1.27 (0.92, 1.74) | 0.148 |
| Cerebrovascular disease |  |  |
| No |  |  |
| Yes | 1.58 (1.10, 2.27) | 0.014 |
| CCI | 1.06 (0.97, 1.14) | 0.186 |
| APS III | 1.00 (0.99, 1.01) | 0.448 |
| SOFA score | 1.04 (0.98, 1.11) | 0.174 |
| Hemoglobin | 0.96 (0.90, 1.03) | 0.226 |
| Respiratory rate | 1.09 (1.03, 1.14) | < 0.001 |
| Specific valve(s) involved |  |  |
| Aortic valve | ref |  |
| Mitral valve | 1.14 (0.83, 1.57) | 0.431 |
| Tricuspid valve | 1.76 (0.81, 3.79) | 0.151 |
| Multiple valves | 1.78 (1.24, 2.54) | 0.002 |
| Type of surgery |  |  |
| Open | ref |  |
| Percutaneous | 3.38 (1.74, 6.56) | < 0.001 |
| Transapical | 1.47 (0.37, 5.89) | 0.587 |
| Use of vasoactive drugs |  |  |
| No |  |  |
| Yes | 1.27 (0.91, 1.76) | 0.155 |
| Use of sedatives |  |  |
| No |  |  |
| Yes | 3.57 (1.93, 6.61) | < 0.001 |
| RRT |  |  |
| No |  |  |
| Yes | 2.38 (1.49, 3.81) | < 0.001 |
| Mechanical ventilation |  |  |
| No |  |  |
| Yes | 2.34 (1.47, 3.73) | < 0.001 |

*POD*, postoperative delirium; *SHR*, stress hyperglycemia ratio; *BMI*, body mass index; *CCI*, Charlson Comorbidity Index; *APS III*, Acute Physiology Score III; *SOFA*, Sequential Organ Failure Assessment; *RRT*, renal replacement therapy.

**Supplementary Table 4.** Comparison of Total POD Assessments Between High- and Low-SHR Groups.

| Postoperative day | Frequency of POD assessments in low-SHR group | Frequency of POD assessments in high-SHR group |
| --- | --- | --- |
| Day 1 | 1.22 ± 1.09 | 1.34 ± 1.04 |
| Day 2 | 1.61 ± 1.52 | 1.83 ± 1.60 |
| Day 3 | 1.32 ± 1.41 | 1.52 ± 1.44 |
| Day 4 | 1.31 ± 1.52 | 1.44 ± 1.37 |
| Day 5 | 1.37 ± 1.48 | 1.39 ± 1.45 |
| Day 6 | 1.47 ± 1.69 | 1.58 ± 1.77 |
| Day 7 | 1.49 ± 1.48 | 1.65 ± 1.70 |

*POD*, postoperative delirium; *SHR*, stress hyperglycemia ratio.

**Supplementary Table 5.** Sensitivity analysis: the relationships between SHR and the risk of POD after including antidiabetic medication in the logistic model.

| Variable | Model 4 | |
| --- | --- | --- |
|  | OR (95% CI) | *P* value |
| SHR | 1.46 (1.02, 2.09) | 0.040 |
| antidiabetic medication |  |  |
| No | ref |  |
| Yes | 0.53 (0.31, 0.91) | 0.021 |

Adjusted for Age, Sex, BMI, Diabetes, Cerebrovascular disease, CCI, APS III, SOFA score, Hemoglobin,

Respiratory rate, Specific valve(s) type, Type of surgery, Use of vasoactive drugs, Use of sedatives, RRT, Mechanical ventilation and antidiabetic medication.

*SHR*, stress hyperglycemia ratio; *POD*, postoperative delirium; *BMI*, body mass index; *CCI*, Charlson Comorbidity

Index; *APS III*, Acute Physiology Score III; *SOFA*, Sequential Organ Failure Assessment; *RRT*, renal replacement

therapy.

**Supplementary Table 6.** Sensitivity analysis: the relationships between SHR and the risk of POD after excluding patients with postoperative sepsis.

| Variable | Model 4 | |
| --- | --- | --- |
|  | OR (95% CI) | *P* value |
| SHR | 1.51 (1.05, 2.19) | 0.028 |

Adjusted for Age, Sex, BMI, Diabetes, Cerebrovascular disease, CCI, APS III, SOFA score, Hemoglobin,

Respiratory rate, Specific valve(s) type, Type of surgery, Use of vasoactive drugs, Use of sedatives, RRT, and

Mechanical ventilation.

*SHR*, stress hyperglycemia ratio; *POD*, postoperative delirium; *BMI*, body mass index; *CCI*, Charlson Comorbidity

Index; *APS III*, Acute Physiology Score III; *SOFA*, Sequential Organ Failure Assessment; *RRT*, renal replacement

therapy.

**Supplementary Table 7.** Sensitivity analysis: the relationships between SHR and the risk of POD after excluding extreme SHR values.

| Variable | Model 4 | |
| --- | --- | --- |
|  | OR (95% CI) | *P* value |
| SHR | 1.66 (1.07, 2.58) | 0.024 |

Adjusted for same factors in eTable 6.

**Supplementary Table 8.** Sensitivity analysis: the relationships between SHR and the risk of POD with SHR categorized into tertiles.

| Variable | Model 4 | |
| --- | --- | --- |
|  | OR (95% CI) | *P* value |
| Tertiles |  |  |
| T1 | 1(ref) |  |
| T2 | 0.95 (0.68, 1.34) | 0.788 |
| T3 | 1.51 (1.10, 2.08) | 0.012 |
| *P* for trend |  | 0.009 |

SHR: T1 (＜0.943), T2 (0.943-1.167), T3 (>1.167).

Adjusted for same factors in eTable 6.

**Supplementary Table 9.** Baseline characteristics between the low-SHR and high-SHR groups.

| Variables | low-SHR (*n* = 1215) | high-SHR (*n* = 615) | *P* value |
| --- | --- | --- | --- |
| Age (years) | 71.00 (62.00, 78.00) | 69.00 (60.00, 77.00) | 0.009 |
| Sex (%) |  |  |  |
| Female | 461 (37.94) | 225 (36.59) | 0.571 |
| Male | 754 (62.06) | 390 (63.41) |  |
| Race (%) |  |  |  |
| Asian | 25 (2.06) | 10 (1.63) | 0.88 |
| Black | 44 (3.62) | 22 (3.58) |  |
| Hispanic | 30 (2.47) | 18 (2.93) |  |
| White | 885 (72.84) | 456 (74.15) |  |
| Other | 231 (19.01) | 109 (17.72) |  |
| BMI (kg/m^2^) | 28.82 (25.24, 32.85) | 28.34 (25.11, 32.70) | 0.177 |
| Myocardial Infarction (%) | 224 (18.44) | 116 (18.86) | 0.825 |
| Congestive heart failure (%) | 581 (47.82) | 316 (51.38) | 0.15 |
| Cerebrovascular disease (%) | 148 (12.18) | 76 (12.36) | 0.913 |
| Hypertension (%) | 709 (58.35) | 328 (53.33) | 0.041 |
| Diabetes mellitus (%) | 377 (31.03) | 138 (22.44) | <0.001 |
| Chronic pulmonary disease (%) | 331 (27.24) | 157 (25.53) | 0.433 |
| Chronic kidney disease (%) | 243 (20.00) | 136 (22.11) | 0.292 |
| Chronic liver disease (%) | 71 (5.84) | 32 (5.20) | 0.574 |
| Malignant cancer (%) | 33 (2.72) | 18 (2.93) | 0.796 |
| CCI | 4.00 (3.00, 6.00) | 4.00 (3.00, 6.00) | 0.312 |
| Hemoglobin (g/dL) | 9.30 (8.10, 10.80) | 9.40 (8.30, 11.20) | 0.029 |
| Sodium (mmol/L) | 138.00 (136.00, 140.00) | 138.00 (136.00, 140.00) | 0.135 |
| Potassium (mmol/L) | 4.30 (4.00, 4.60) | 4.30 (3.90, 4.60) | 0.393 |
| BUN (mg/dL) | 16.00 (13.00, 23.00) | 17.00 (13.00, 24.00) | 0.129 |
| Creatinine (mg/dL) | 0.90 (0.70, 1.10) | 0.90 (0.80, 1.20) | <0.001 |
| HR (beats/minute) | 79.81 (74.82, 85.43) | 80.57 (74.78, 87.13) | 0.12 |
| SBP (mmHg) | 110.42 (105.39, 116.35) | 110.25 (103.72, 116.73) | 0.491 |
| DBP (mmHg) | 56.33 (51.91, 61.22) | 57.07 (52.20, 61.85) | 0.052 |
| MAP (mmHg) | 73.29 (69.52, 77.41) | 73.82 (69.58, 78.33) | 0.094 |
| Respiratory rate (beats/minute) | 17.62 (16.24, 19.39) | 18.24 (16.50, 20.10) | <0.001 |
| SpO2 (%) | 97.90 (96.97, 98.81) | 97.79 (96.66, 98.81) | 0.099 |
| APS Ⅲ | 35.00 (27.00, 46.00) | 37.00 (29.00, 51.00) | 0.002 |
| SOFA score | 6.00 (4.00, 8.00) | 6.00 (4.00, 8.00) | 0.775 |
| Specific valve(s) involved (%) |  |  |  |
| Aortic valve | 664 (54.65) | 337 (54.80) | 0.998 |
| Mitral valve | 349 (28.72) | 175 (28.46) |  |
| Tricuspid valve | 31 (2.55) | 15 (2.44) |  |
| Multiple valves | 171 (14.07) | 88 (14.31) |  |
| Type of surgery (%) |  |  |  |
| Open | 1172 (96.46) | 561 (91.22) | <0.001 |
| Percutaneous | 36 (2.96) | 48 (7.80) |  |
| Transapical | 7 (0.58) | 6 (0.98) |  |
| Use of vasoactive drugs (%) | 888 (73.09) | 420 (68.29) | 0.032 |
| Use of sedatives (%) | 1087 (89.47) | 528 (85.85) | 0.023 |
| Mechanical ventilation (%) | 1001 (82.39) | 487 (79.19) | 0.097 |
| RRT (%) | 63 (5.19) | 52 (8.46) | 0.006 |

low-SHR: SHR < 1.164; high-SHR: SHR ≥ 1.164.

*BMI*, body mass index; *CCI*, Charlson Comorbidity Index; *BUN*, blood urea nitrogen; *SHR*, stress hyperglycemia ratio; *HR*, heart rate; *SBP*, systolic blood pressure; *DBP*, diastolic blood pressure; *MAP*, mean arterial pressure; *SpO2*, peripheral oxygen saturation; *APS Ⅲ*, Acute Physiology Score III; *SOFA*, Sequential Organ Failure Assessment; *RRT*, renal replacement therapy.

**Supplementary Figure 1.** Daily frequency of POD assessments during the first 7 postoperative days.


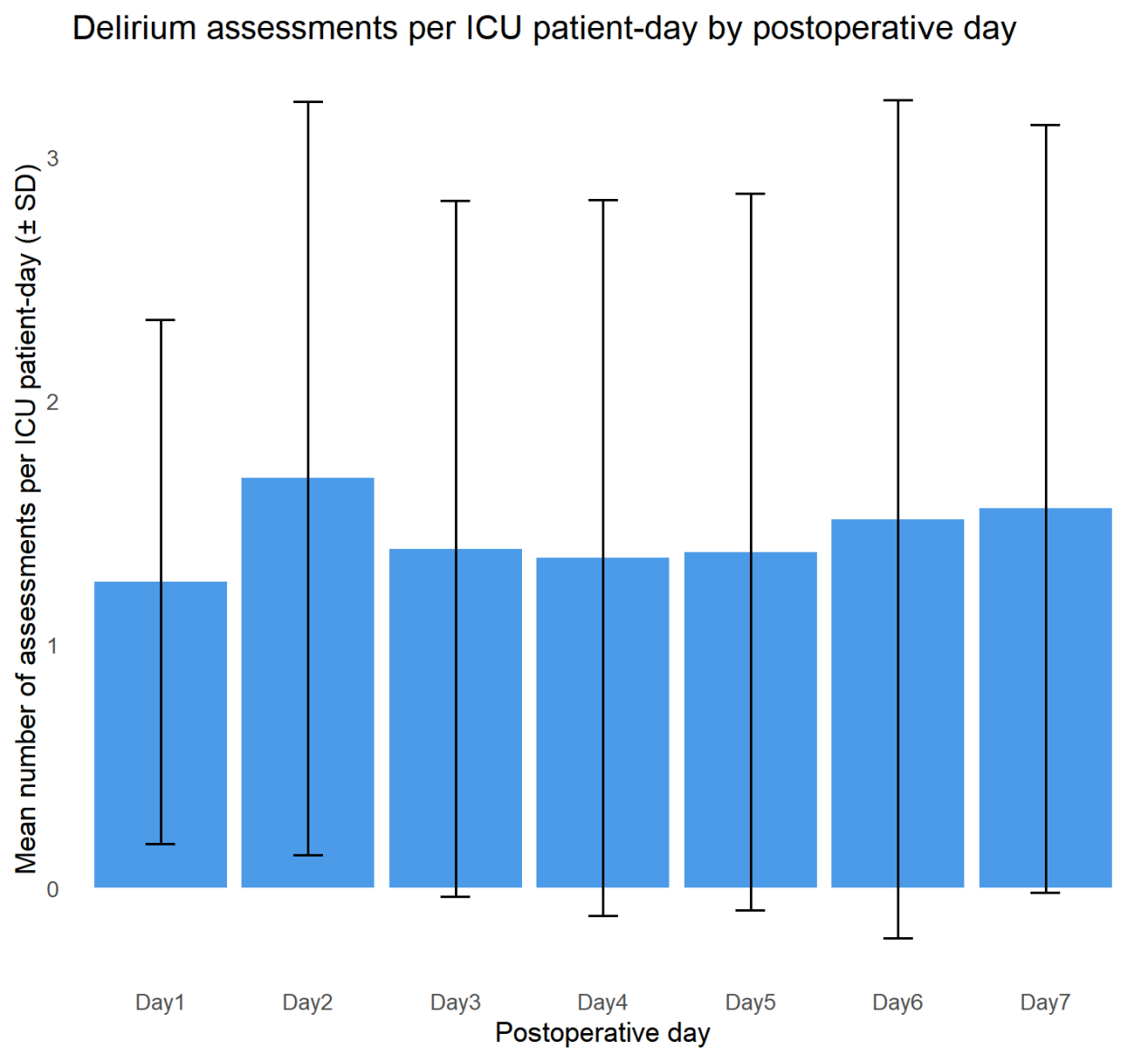

Supplement: Supplementary file 1 — Supplementary Material 1 [file 41598_2026_41714_MOESM1_ESM.docx]
